# The promising role of risk scoring system for Cushing syndrome: Time to reconsider current screening recommendations

**DOI:** 10.3389/fendo.2022.1075785

**Published:** 2022-11-22

**Authors:** CE. Lam-Chung, D. Cuevas-Ramos

**Affiliations:** ^1^ Department of Endocrinology and Metabolism, Complejo Hospitalario Dr. Manuel Amador Guerrero, Colón, Panama; ^2^ Neuroendocrinology Clinic, Department of Endocrinology and Metabolism, Instituto Nacional de Ciencias Médicas y Nutrición Salvador Zubirán, Mexico City, Mexico

**Keywords:** cortisol, ACTH, corticotropin, pituitary adenoma, obesity

## Abstract

Despite the current screening approach for Cushing syndrome (CS), delayed diagnosis is common due to broad spectrum of presentation, poor discriminant symptoms featured in diabetes and obesity, and low clinical index of suspicion. Even if initial tests are recommended to screen CS, divergent results are not infrequent. As global prevalence of type 2 diabetes and obesity increases, CS may not be frequent enough to back routine screening to avoid false-positive results. This represents a greater challenge in countries with limited health resources. The development of indexes incorporates clinical features and biochemical data that are largely used to provide a tool to predict the presence of disease. In clinical endocrinology, indexes have been used in Graves’ ophthalmology, hirsutism, and hypothyroidism. The use of clinical risk scoring system may assist clinicians in discriminating CS in the context of at-risk populations and, thus, may provide a potential intervention to decrease time to diagnosis. Development and validation of clinical model to estimate pre-test probability of CS in different geographic source population may help to establish regional prediction model for CS. Here, we review on the latest progress in clinical risk scoring system for CS and attempt to raise awareness for the use, validation, and/or development of clinical risk scores in CS.

## Introduction

Cushing syndrome (CS) is caused by continued exposure to excess of either endogenous cortisol or exogenous glucocorticoids. Endogenous CS results from improper hypercortisolism due to either adrenocorticotropin (ACTH) hypersecretion or autonomous adrenal cortisol hypersecretion (1, 2). It is an infrequent endocrine disease that poses diagnostic challenges even by attendant endocrinologists (3, 4). To complicate matters, patients may present with few clinical or nonspecific features (1, 5). Therefore, it is very challenging to diagnose CS in the early stages or in cyclic CS (6–8).

The Endocrine Society’s Clinical Practice Guideline for the diagnosis of CS recommends that exogenous glucocorticoids intake should be rule out before performing screening tests (1, 9). It also states that hypercortisolism should be tested in patients in whom a diagnosis is most likely with signs and symptoms that include weight gain, abnormal glucose tolerance, and hypertension (1, 9, 10). Global prevalence of type 2 diabetes (T2D) and obesity is becoming more common, and these, in turn, are commonly associated with mild increased cortisol levels (11, 12). Moreover, other conditions with secondarily activated hypothalamic–pituitary–adrenal such as depression, alcohol dependence, and glucocorticoid resistance offer even more difficulties in discriminating CS (1, 13). Even in highly specialized center, half of the patients submitted for screening of CS did not belong to the recommended groups for screening (9). As the lifetime experience to diagnose CS for a physician is very low (14, 15), we can only speculate that the screening tests for hypercortisolism might be unnecessary used, leading to false-positive results. Unaided clinical diagnosis may be difficult even for seasoned endocrinologists, especially in patients with no clear-cut features of CS, no use of exogenous glucocorticoids, and associated with mild hypercortisolism conditions.

To reduce unnecessary screening and shorten the time of diagnosis, numerous attempts have been made including clinical scores (16–18), automated face recognition (19–21), radiological or clinical assessments of skin thickness (22, 23), the quantification of facial plethora with near-infrared imaging (24), and, more recently, changes in white blood cell count (25, 26). This mini-review focuses on the latest progress in clinical risk scoring system for CS. We attempt to raise awareness for the use, validation, and/or development of clinical risk scores in CS.

## Method for a systematic review

A systematic search was conducted in PubMed, Medline, EBSCO, Web of Science, ScienceDirect, Scopus, and OVID, for articles that developed and/or validated clinical risk score for the pre-test probability of CS. All epidemiological, cross-sectional, cohort, retrospective, longitudinal, observational, comparative, case-control, and case-report studies were considered, which contained the following keywords or MeSH terms: “cortisol”, “ACTH”, “corticotropin”, “pituitary adenoma”, and “Cushing syndrome”. Articles written in English or Spanish were included.

## Difficulties with current screening tests for CS

Simple screening tests to discriminate CS are needed as early diagnosis can prevent associated comorbidities and mortality (27, 28). Moreover, the appropriate use of testing to screen patient-at-risk is crucial because delayed diagnosis may be associated with symptoms after treatment (29). CS is an infrequent disease that has a low prevalence so its screening test must have a high specificity to avoid false-positive results (30, 31). Current clinical guidelines outlined by the evidence-based 2008 Endocrine Society suggest testing with one of the following first-line tests: late-night salivary cortisol (LNSC) (two measurements), 24-h urinary free cortisol (UFC) excretion (two measurements), or the overnight 1-mg dexamethasone suppression test (DST). The performance of these first-line screening tests in usual clinical practice is uncertain as none of them have ideal sensitivity or specificity (32, 33). **Table 1** shows the diagnostic criteria and sensitivities and specificities for these tests. Consequently, clinicians must individualize the choice for the initial test to reduce false-positive results. There is a need to increase clinician awareness concerning the need of complementary and individualized tests to determine CS diagnosis. LNSC assay usefulness for screening is based on the lack of circadian rhythm in CS (1, 44). Its use has increased overtime (45). One of the challenges of LNSC is on its cutoff to interpretate the results. Thresholds and accuracy vary widely, perhaps, due to assay and/or collection differences (40, 41). Sensitivities and specificities ranges from 83% to 100% and 84% to 97%, respectively (39–43). Despite its advantage to be non-invasiveness, LNSC is not useful to diagnose CS in patients with irregular sleep schedule, night worker, and in older men with comorbidities such as T2D and/or hypertension (1, 46). Appropriate assay-specific, age-specific values, sex, age, and other medical conditions on LNSC have not been fully characterized (1, 47).

**Table 1:** Sensitivity and specificity and likelihood ratio of screening tests for Cushing syndrome (34–43).

Variable	Sensitivity (%)	Specificity (%)	+LHR (95% CI)	−LHR (95% CI)
1-mg DST	91–97	87–94	16.4 (9.3–28.8)	0.06 (0.03–0.14)
24-h UFC	90–98	45–95	10.6 (5.5–20.5)	0.16 (0.08–0.33)
LNSC	83–100	84–97	9.5 (1.7–54.1)	0.09 (0.03–0.28)

DST, dexamethasone test; UFC, urinary free cortisol; LNSC, late-night salivary cortisol; +LHR, positive likelihood ratio; −LHR, negative likelihood ratio; CI, confidence interval.

Twenty-four–hour UFC is a reliable first-line screening test with sensitivity and specificity of up to 98% and 92%, respectively (37, 38). Current guidelines suggest performing at least 24-h UFC determinations, and patients can be considered to have CS if UFC is above more than three times the upper limit of normal together with another abnormal first-line screening test (1). One of the main issues with UFC is its limited utility in subclinical hypercortisolism, resulting in a false-negative result (1). Regardless of some limitation for its interpretation, UFC has a superior diagnostic performance with a likelihood ratio of 10.6 (95% CI, 5.5–20.5) for an abnormal result and a likelihood ratio for CS of 0.16 (95% CI, 0.08–0.33) for a normal result (36).

Overnight 1-mg DST is used to discriminate patients if the patients have CS of any etiology from patients who do not have it (1). Broadly speaking, overnight 1-mg DST remains the screening test of choice, but its sensitivity and specificity depend on the threshold of plasma cortisol used (37, 38, 48). False-positive results can occur in patients taking oral estrogens that increase in corticosteroid-binding globulin or any medications that modify the metabolism of dexamethasone (1, 49). False-negative results can be seen on patients with decreased metabolism of dexamethasone secondary to drugs, liver, or kidney failure (50).

## Clinical scores to identify individuals at increased risk of CS

CS is an infrequent disease, and few centers see sufficiently large numbers of patients to collect sufficient data to obtain reliable estimates of predictors within a short period of time (30, 31). Correspondingly, there are few published studies that attempt to weight each clinical variable (16–18). Until now, few diagnostic scores for CS have been developed to identify which patients should be screened for CS (16–18). First developed in 1964 by Nugent et al., a clinical score based on 19 simple clinical and laboratory data associated with hypercortisolism was used to increase the diagnostics usefulness (16). However, only half of the patients with suspected CS could be diagnosed, correctly lacking its clinical usefulness (**Table 2**). León-Justel et al. proposed a risk score based on clinical signs and one biochemical parameter that included 353 at-risk patients from 13 different hospitals across Spain (17). Screening was considered having at least two of the risk factors for CS: high blood pressure (defined as taking two or more drugs and having a systolic blood pressure over 140 mmHg and/or a diastolic blood pressure over 90 mmHg), obesity (body mass index >30), uncontrolled T2D (HbA1c >7.0%), osteoporosis (T-score ≥−2.5 SD), and virilization syndrome (hirsutism) with menstrual disorders. Biochemical data including LNSC and the low-dose DST were used. Risk score for CS included osteoporosis, dorsocervical fat pad, and muscular atrophy and LNSC levels. The researchers developed an equation to determine the risk of CS with sensitivity and specificity for this model being 96% and 83%, respectively. Although this study was a good start to identify key predictors for CS, it included a biochemical test, making it inappropriate for its applicability in an exclusive clinical estimate of the probability of CS before testing (**Table 2**). Moreover, the included clinical signs are already well known to be associated with endogenous hypercortisolism, and LNSC (included in the equation) is already a screening test for CS (1). Finally, several methodological flaws limit its reliability (51).

**Table 2:** Cushing syndrome clinical risk scores.

Score	Information used	Statistics used
Nugent et al. (16)	19 signs and symptoms related with CS (see text)	Probability of 01 or less (very unlikely); between 0.01 and 0.1 (unlikely); from 0.1 to 0.9 (uncertain); between 0.9 and 0.99 (likely); 0.99 or more (very likely)
Leon-Justel et al. (17)	Hypertension, diabetes, osteoporosis, obesity, virilization, and menstrual irregularities	Sensitivity, 96% Specificity, 83% AUC = 0.68 and AUC = 0.93 including LNSC
Parasiliti-Caprino et al. (18)	Age, facial fullness and plethora, proximal muscle atrophy, hirsutism and/or seborrhea, absence of obesity, hypertension, diabetes, and bone mineral density	Sensitivity, 96.2% Specificity, 82.9% AUC = 0.87

AUC, area under the curve.

Parasiliti-Caprino et al. developed and internally validated the “Cushing score” to discriminate between a low- and high-pretest probability of CS (18). The researchers included 150 CS cases and 300 controls from five endocrinology centers in Italy. Baseline characteristics associated with CS were collected, including muscle wasting (proximal muscle atrophy and proximal muscle weakness), skin changes (easy bruisability, facial plethora, hirsutism, purple striae, and/or seborrhea), atypical fat distribution (central adiposity, dorsocervical fat pad, and facial fullness), bone mineral loss (osteopenia or osteoporosis), cardiometabolic alterations (diabetes, dyslipidemia, hypertension, and obesity), and psychiatric symptoms. The prediction model for CS was built from a multivariable logistic regression that included key features associated with clinical impression of hypercortisolism. Predictive variables included age [odds ratio (OR), 3.15; 95% CI, 1.34–7.42; *P* = 0.009 for age 40-59 years; OR, 7.35; 95% CI, 2.79–19.37; *P* < 0.001 for age < 40 years], facial fullness (OR, 2.13; 95% CI, 1.16–3.93; *P* = 0.015), facial plethora (OR, 1.98; 95% CI, 1.04–3.77; *P* = 0.038), proximal muscle atrophy (OR, 2.46; 95% CI, 1.24–4.88; *P* = 0.010), hirsutism and/or seborrhea (OR, 1.91; 95% CI, 1.06–3.41; *P* = 0.030), absence of obesity (OR, 5.93; 95% CI, 3.27–10.73; *P* < 0.001), hypertension (OR, 3.36; 95% CI, 1.81–6.21; *P* < 0.001), diabetes (OR, 1.87; 95% CI, 0.98–3.57; *P* = 0.059), and bone mineral density (OR, 2.35; 95% CI, 1.14–4.86, *P* = 0.021 for osteopenia; OR, 5.13; 95% CI, 2.39–11.02; *P* < 0.001 for osteoporosis). Receiver operating characteristic curve analysis and area under the curve (AUC) were used, showing a high predictive performance (0.873). In addition, internal validation was conducted, showing an AUC of 0.841. The prediction model “Cushing score” was a 17.5-point scale classified as follows: low-risk class (score value, ≤5.5; probability of disease, 0.8%), an intermediate-low-risk class (score value, 6–8.5; probability of disease, 2.7%), an intermediate-high-risk class (score value, 9–11.5; probability of disease, 18.5%), and a high-risk class (score value, 12.0–17.5; probability of disease, 72.5%).

Given the heterogenous nature of CS (1, 16), whether it is possible to accurately identify patients at risk using a model remains to be determined. As global prevalence of T2D and obesity increases (52, 53). CS may not be frequent enough to back routine screening to avoid false-positive results. Those clinical scores are summarized in **Table 2**.

## Clinical risk scoring system in the era of high volume of scientific knowledge

Risk scores are useful to stratify a population for targeted screening. Data from risk factors are used to calculate an individual’s score (54). In clinical endocrinology, risk scores have been used in Graves’ ophthalmology (55), hirsutism (56), hypothyroidism (57), and even in CS (16). Clinically relevant information derived from research assists clinicians to avoid unnecessary diagnosis tests or treatment (58). Discrimination of CS in the absence of clear-cut features is challenging, especially in the context of the ever-increasing prevalence of T2D, obesity, and depression (52, 53, 59). Time to diagnosis of CS remains to be delayed as its signs and symptoms overlap with common diseases including metabolic syndrome and polycystic ovary syndrome (15, 27). Moreover, the time taken to diagnose CS differs according to its subtypes and geographic regions (27). A standardized approach with a clinical score system based on applied evidence might decrease the time of diagnosis, and a timely diagnosis of CS decreases its morbidity and chronic sequalae (27, 28, 59).

One of the current challenges of CS is it rareness and the absence of lead symptoms (13, 30, 31). Moreover, different definitions of first symptoms of CS might explain why time diagnosis varies (27). There are several well-established characteristic phenotypes associated with chronic hypercortisolism such as weight gain, menstrual irregularities, hirsutism, muscle weakness, bruisability, skin atrophy, and buffalo hump; however, the independent contribution of each factor is not well understood or defined (60). In addition, comorbidities including hypertension, hypercholesterolemia, T2D, and osteoporosis are likely to be present in patients with hypercortisolism, but systematic screening recommendation for CS is questioned (9, 61–64). The combination of these clinical data into tools to estimate the risk and, therefore, to screen for CS, is still in its infancy (15). The development of an algorithm for predicting whom to screen CS could assist clinicians to consider it with the ultimate goal of reducing morbidity and mortality.

## Discussion

Because the prevalence of endogenous hypercortisolism disease is increasing because of metabolic and chronic diseases such as T2D, obesity, metabolic syndrome, and depression, there might be a need for the use of diagnostic scores (preferably based only on clinical signs) to guide physicians the use of initial screening test. One of the objectives in this review is to raise awareness in the medical community regarding the use, validation, and/or development of clinical risk scores in CS.

## Conclusion

CS diagnoses are still delayed, increasing the danger of poor prognosis. Because of a high prevalence of T2D, obesity, and other comorbidities, it is important to build and validate a simple risk score prediction for CS based on easily acquired clinical variables to achieve potential risk reduction and healthcare costs, diagnostic tests, and treatment of comorbidities.

## Author contributions

Both authors did data collection, writing, and critical revision of the article. All authors contributed to the article and approved the submitted version.

## Conflict of interest

The authors declare that the research was conducted in the absence of any commercial or financial relationships that could be construed as a potential conflict of interest.

## Publisher’s note

All claims expressed in this article are solely those of the authors and do not necessarily represent those of their affiliated organizations, or those of the publisher, the editors and the reviewers. Any product that may be evaluated in this article, or claim that may be made by its manufacturer, is not guaranteed or endorsed by the publisher.

## References

[1] Nieman LK Biller BM Findling JW Newell-Price J Savage MO Stewart PM . The diagnosis of cushing’s syndrome: an endocrine society clinical practice guideline. J Clin Endocrinol Metab (2008) 93(5):1526–40. doi: 10.1210/jc.2008-0125 PMC2386281 18334580

[2] Hasenmajer V Sbardella E Sciarra F Minnetti M Isidori AM Venneri MA . The immune system in cushing’s syndrome. Trends Endocrinol metabolism: TEM. (2020) 31(9):655–69. doi: 10.1016/j.tem.2020.04.004 32387195

[3] Findling JW Raff H . DIAGNOSIS OF ENDOCRINE DISEASE: Differentiation of pathologic/neoplastic hypercortisolism (Cushing’s syndrome) from physiologic/non-neoplastic hypercortisolism (formerly known as pseudo-cushing’s syndrome). Eur J Endocrinol (2017) 176(5):R205–r16. doi: 10.1530/EJE-16-0946 28179447

[4] Kreitschmann-Andermahr I Psaras T Tsiogka M Starz D Kleist B Siegel S . From first symptoms to final diagnosis of cushing’s disease: experiences of 176 patients. Eur J endocrinol (2015) 172(3):285–9. doi: 10.1530/EJE-14-0766 25501963

[5] Sharma ST Nieman LK Feelders RA . Cushing’s syndrome: epidemiology and developments in disease management. Clin Epidemiol (2015) 7:281–93. doi: 10.2147/CLEP.S44336 PMC4407747 25945066

[6] Cipoli DE Martinez EZ Castro M Moreira AC . Clinical judgment to estimate pretest probability in the diagnosis of cushing’s syndrome under a Bayesian perspective. Arquivos brasileiros endocrinologia e metabologia. (2012) 56(9):633–7. doi: 10.1590/S0004-27302012000900006 23329186

[7] Savas M Mehta S Agrawal N van Rossum E Feelders RA . Approach to the patient: Diagnosis of cushing’s syndrome. J Clin Endocrinol Metab (2022) dgac492. doi: 10.1210/clinem/dgac492 PMC9681610 36036941

[8] Świątkowska-Stodulska R Berlińska A Stefańska K Kłosowski P Sworczak K . Cyclic cushing’s syndrome - a diagnostic challenge. Front endocrinol (2021) 12:658429. doi: 10.3389/fendo.2021.658429 PMC8101412 33967962

[9] Braun LT Vogel F Zopp S Marchant Seiter T Rubinstein G Berr CM . Whom should we screen for cushing syndrome? the endocrine society practice guideline recommendations 2008 revisited. J Clin Endocrinol Metab (2022) 107(9):e3723–e30. doi: 10.1210/clinem/dgac379 PMC9387700 35730067

[10] Lacroix A Feelders RA Stratakis CA Nieman LK . Cushing’s syndrome. Lancet (London England) (9996) 2015:913–27:386. doi: 10.1016/S0140-6736(14)61375-1 26004339

[11] Scaroni C Albiger NM Palmieri S Iacuaniello D Graziadio C Damiani L . Approach to patients with pseudo-cushing’s states. Endocrine connections (2020) 9(1):R1–r13. doi: 10.1530/EC-19-0435 31846432 PMC6993268

[12] Li D El Kawkgi OM Henriquez AF Bancos I . Cardiovascular risk and mortality in patients with active and treated hypercortisolism. Gland surgery (2020) 9(1):43–58. doi: 10.21037/gs.2019.11.03 32206598 PMC7082278

[13] Debono M Newell-Price JD . Cushing’s syndrome: Where and how to find it. Front hormone Res (2016) 46:15–27. doi: 10.1159/000443861 27211887

[14] Aron DC . Cushing’s syndrome: why is diagnosis so difficult? Rev endocrine Metab Disord (2010) 11(2):105–16. doi: 10.1007/s11154-010-9127-3 20217244

[15] Braun LT Riester A Oßwald-Kopp A Fazel J Rubinstein G Bidlingmaier M . Toward a diagnostic score in cushing’s syndrome. Front endocrinol (2019) 10:766. doi: 10.3389/fendo.2019.00766 PMC6856055 31787931

[16] Nugent CA Warner HR Dunn JT Tyler FH . Probability theory in the diagnosis of cushing’s syndrome. J Clin Endocrinol Metab (1964) 24:621–7. doi: 10.1210/jcem-24-7-621 14212081

[17] León-Justel A Madrazo-Atutxa A Alvarez-Rios AI Infantes-Fontán R Garcia-Arnés JA Lillo-Muñoz JA . A probabilistic model for cushing’s syndrome screening in At-risk populations: A prospective multicenter study. J Clin Endocrinol Metab (2016) 101(10):3747–54. doi: 10.1210/jc.2016-1673 27490917

[18] Parasiliti-Caprino M Bioletto F Frigerio T D’Angelo V Ceccato F Ferraù F . A new clinical model to estimate the pre-test probability of cushing’s syndrome: The cushing score. Front endocrinol (Lausanne) (2021) 12:747549. doi: 10.3389/fendo.2021.747549 34675882 PMC8524092

[19] Learned-Miller E Lu Q Paisley A Trainer P Blanz V Dedden K . Detecting acromegaly: screening for disease with a morphable model. Med image computing computer-assisted intervention: MICCAI Int Conf Med Image Computing Computer-Assisted Intervention (2006) 9(Pt 2):495–503. doi: 10.1007/11866763_61 17354809

[20] Schneider HJ Kosilek RP Günther M Roemmler J Stalla GK Sievers C . A novel approach to the detection of acromegaly: accuracy of diagnosis by automatic face classification. J Clin Endocrinol Metab (2011) 96(7):2074–80. doi: 10.1210/jc.2011-0237 21508144

[21] Kosilek RP Schopohl J Grunke M Reincke M Dimopoulou C Stalla GK . Automatic face classification of cushing’s syndrome in women - a novel screening approach. Exp Clin Endocrinol diabetes: Off journal German Soc Endocrinol [and] German Diabetes Assoc (2013) 121(9):561–4. doi: 10.1055/s-0033-1349124 23864496

[22] Ferguson JK Donald RA Weston TS Espiner EA . Skin thickness in patients with acromegaly and cushing’s syndrome and response to treatment. Clin endocrinol (1983) 18(4):347–53. doi: 10.1111/j.1365-2265.1983.tb00578.x 6872269

[23] Corenblum B Kwan T Gee S Wong NC . Bedside assessment of skin-fold thickness. a useful measurement for distinguishing cushing’s disease from other causes of hirsutism and oligomenorrhea. Arch Internal Med (1994) 154(7):777–81. doi: 10.1001/archinte.1994.00420070099011 8147682

[24] Afshari A Ardeshirpour Y Lodish MB Gourgari E Sinaii N Keil M . Facial plethora: Modern technology for quantifying an ancient clinical sign and its use in cushing syndrome. J Clin Endocrinol Metab (2015) 100(10):3928–33. doi: 10.1210/jc.2015-2497 PMC4596033 26301943

[25] Paja M Merlo I Rodríguez-Soto J Cruz-Iglesias E Moure MD Elías C . White blood cell count: a valuable tool for suspecting cushing’s syndrome. J Endocrinol Invest (2022). doi: 10.1007/s40618-022-01892-6 35943722

[26] Detomas M Altieri B Chifu I Remde H Zhou X Landwehr LS . Subtype-specific pattern of white blood cell differential in endogenous hypercortisolism. Eur J endocrinol (2022) 187(3):439–49. doi: 10.1530/EJE-22-0211 35900357

[27] Rubinstein G Osswald A Hoster E Losa M Elenkova A Zacharieva S . Time to diagnosis in cushing’s syndrome: A meta-analysis based on 5367 patients. J Clin Endocrinol Metab (2020) 105(3):e12–e22. doi: 10.1210/clinem/dgz136 31665382

[28] Javanmard P Duan D Geer EB . Mortality in patients with endogenous cushing’s syndrome. Endocrinol Metab Clinics North America (2018) 47(2):313–33. doi: 10.1016/j.ecl.2018.02.005 29754634

[29] Valassi E Chiodini I Feelders RA Andela CD Abou-Hanna M Idres S . Unmet needs in cushing’s syndrome: the patients’ perspective. Endocr Connect (2022) 11(7):e220027. doi: 10.1530/EC-22-0027 35904235 PMC9254293

[30] Steffensen C Bak AM Rubeck KZ Jørgensen JO . Epidemiology of cushing’s syndrome. Neuroendocrinology. (2010) 92 Suppl 1:1–5. doi: 10.1159/000314297 20829610

[31] Valassi E Santos A Yaneva M Tóth M Strasburger CJ Chanson P . The European registry on cushing’s syndrome: 2-year experience. Baseline demographic Clin characteristics Eur J endocrinol (2011) 165(3):383–92. doi: 10.1530/EJE-11-0272 21715416

[32] Ceccato F Boscaro M . Cushing’s syndrome: Screening and diagnosis. High Blood Pressure Cardiovasc prevention: Off J Ital Soc Hypertension (2016) 23(3):209–15. doi: 10.1007/s40292-016-0153-4 27160717

[33] Baid SK Rubino D Sinaii N Ramsey S Frank A Nieman LK . Specificity of screening tests for cushing’s syndrome in an overweight and obese population. J Clin Endocrinol Metab (2009) 94(10):3857–64. doi: 10.1210/jc.2008-2766 PMC2758724 19602562

[34] Isidori AM Kaltsas GA Mohammed S Morris DG Jenkins P Chew SL . Discriminatory value of the low-dose dexamethasone suppression test in establishing the diagnosis and differential diagnosis of cushing’s syndrome. J Clin Endocrinol Metab (2003) 88(11):5299–306. doi: 10.1210/jc.2003-030510 14602765

[35] Meikle AW . Dexamethasone suppression tests: usefulness of simultaneous measurement of plasma cortisol and dexamethasone. Clin endocrinol (1982) 16(4):401–8. doi: 10.1111/j.1365-2265.1982.tb00733.x 7094363

[36] Elamin MB Murad MH Mullan R Erickson D Harris K Nadeem S . Accuracy of diagnostic tests for cushing’s syndrome: a systematic review and metaanalyses. J Clin Endocrinol Metab (2008) 93(5):1553–62. doi: 10.1210/jc.2008-0139 18334594

[37] Ceccato F Barbot M Zilio M Frigo AC Albiger N Camozzi V . Screening tests for cushing’s syndrome: Urinary free cortisol role measured by LC-MS/MS. J Clin Endocrinol Metab (2015) 100(10):3856–61. doi: 10.1210/jc.2015-2507 26274344

[38] Isidori AM Graziadio C Paragliola RM Cozzolino A Ambrogio AG Colao A . The hypertension of cushing’s syndrome: controversies in the pathophysiology and focus on cardiovascular complications. J hypertension (2015) 33(1):44–60. doi: 10.1097/HJH.0000000000000415 PMC4342316 25415766

[39] Antonelli G Ceccato F Artusi C Marinova M Plebani M . Salivary cortisol and cortisone by LC-MS/MS: validation, reference intervals and diagnostic accuracy in cushing’s syndrome. Clinica chimica acta; Int J Clin Chem (2015) 451(Pt B):247–51. doi: 10.1016/j.cca.2015.10.004 26449783

[40] Ceccato F Barbot M Zilio M Ferasin S Occhi G Daniele A . Performance of salivary cortisol in the diagnosis of cushing’s syndrome, adrenal incidentaloma, and adrenal insufficiency. Eur J endocrinol (2013) 169(1):31–6. doi: 10.1530/EJE-13-0159 23610124

[41] Deutschbein T Broecker-Preuss M Flitsch J Jaeger A Althoff R Walz MK . Salivary cortisol as a diagnostic tool for cushing’s syndrome and adrenal insufficiency: improved screening by an automatic immunoassay. Eur J endocrinol (2012) 166(4):613–8. doi: 10.1530/EJE-11-0945 22214924

[42] Erickson D Singh RJ Sathananthan A Vella A Bryant SC . Late-night salivary cortisol for diagnosis of cushing’s syndrome by liquid chromatography/tandem mass spectrometry assay. Clin endocrinol (2012) 76(4):467–72. doi: 10.1111/j.1365-2265.2011.04239.x 21955126

[43] Raff H . Cushing’s syndrome: diagnosis and surveillance using salivary cortisol. Pituitary. (2012) 15(1):64–70. doi: 10.1007/s11102-011-0333-0 21833616

[44] Boscaro M Arnaldi G . Approach to the patient with possible cushing’s syndrome. J Clin Endocrinol Metab (2009) 94(9):3121–31. doi: 10.1210/jc.2009-0612 19734443

[45] Bäcklund N Brattsand G Israelsson M Ragnarsson O Burman P Edén Engström B . Reference intervals of salivary cortisol and cortisone and their diagnostic accuracy in cushing’s syndrome. Eur J endocrinol (2020) 182(6):569–82. doi: 10.1530/EJE-19-0872 32213657

[46] Liu H Bravata DM Cabaccan J Raff H Ryzen E . Elevated late-night salivary cortisol levels in elderly male type 2 diabetic veterans. Clin endocrinol (2005) 63(6):642–9. doi: 10.1111/j.1365-2265.2005.02395.x 16343098

[47] Fleseriu M . Salivary cortisol in the diagnosis of cushing syndrome, always more than one! J Endocrine Soc (2020) 4(10):bvaa109. doi: 10.1210/jendso/bvaa109 32939437 PMC7480955

[48] Pecori Giraldi F Ambrogio AG De Martin M Fatti LM Scacchi M Cavagnini F . Specificity of first-line tests for the diagnosis of cushing’s syndrome: assessment in a large series. J Clin Endocrinol Metab (2007) 92(11):4123–9. doi: 10.1210/jc.2007-0596 17698908

[49] Valassi E Swearingen B Lee H Nachtigall LB Donoho DA Klibanski A . Concomitant medication use can confound interpretation of the combined dexamethasone-corticotropin releasing hormone test in cushing’s syndrome. J Clin Endocrinol Metab (2009) 94(12):4851–9. doi: 10.1210/jc.2009-1500 PMC2795659 19850679

[50] Gilbert R Lim EM . The diagnosis of cushing’s syndrome: an endocrine society clinical practice guideline. Clin biochemist Rev (2008) 29(3):103–6. doi: 10.1210/jc.2008-0125 PMC2605410 19107223

[51] Collins GS Le Manach Y . Letter to the Editor: Models developed using small datasets should be appropriately evaluated. J Clin Endocrinol Metab (2016) 101(11):L104–L5. doi: 10.1210/jc.2016-2934 27809722

[52] Cooper AJ Gupta SR Moustafa AF Chao AM . Sex/Gender differences in obesity prevalence, comorbidities, and treatment. Curr Obes Rep (2021) 10(4):458–66. doi: 10.1007/s13679-021-00453-x 34599745

[53] Ferrari AJ Santomauro DF Herrera AMM Shadid J Ashbaugh C Erskine HE . Global, regional, and national burden of 12 mental disorders in 204 countries and territories, 1990-2019: a systematic analysis for the global burden of disease study 2019. Lancet Psychiatry (2022) 9(2):137–50. doi: 10.1016/S2215-0366(21)00395-3 PMC8776563 35026139

[54] Davies MJ Gray LJ Troughton J Gray A Tuomilehto J Farooqi A . Developing the risk score. 2017. In: A community-based primary prevention programme for type 2 diabetes mellitus integrating identification and lifestyle intervention for prevention: a cluster randomised controlled trial. Available at: https://www.ncbi.nlm.nih.gov/books/NBK409312/. 28121091

[55] Stan MN Garrity JA Bahn RS . The evaluation and treatment of graves ophthalmopathy. Med Clinics North America (2012) 96(2):311–28. doi: 10.1016/j.mcna.2012.01.014 PMC3898790 22443978

[56] Sachdeva S . Hirsutism: evaluation and treatment. Indian J Dermatol (2010) 55(1):3–7. doi: 10.4103/0019-5154.60342 20418968 PMC2856356

[57] Kalra S Khandelwal SK Goyal A . Clinical scoring scales in thyroidology: A compendium. Indian J Endocrinol Metab (2011) 15(Suppl 2):S89–94. doi: 10.4103/2230-8210.83332 PMC3169861 21966660

[58] Galvao MC Ricarte IL Grad RM Pluye P . The clinical relevance of information index (CRII): assessing the relevance of health information to the clinical practice. Health Inf libraries J (2013) 30(2):110–20. doi: 10.1111/hir.12021 23692452

[59] Sharma ST Nieman LK Feelders RA . Comorbidities in cushing’s disease. Pituitary. (2015) 18(2):188–94. doi: 10.1007/s11102-015-0645-6 PMC4374115 25724314

[60] Nieman LK . Cushing’s syndrome: update on signs, symptoms and biochemical screening. Eur J Endocrinol (2015) 173(4):M33–8. doi: 10.1530/EJE-15-0464 PMC4553096 26156970

[61] Tabarin A Perez P . Pros and cons of screening for occult cushing syndrome. Nat Rev Endocrinol (2011) 7(8):445–55. doi: 10.1038/nrendo.2011.51 21423244

[62] Shimon I . Screening for cushing’s syndrome: is it worthwhile? Pituitary (2015) 18(2):201–5. doi: 10.1007/s11102-015-0634-9 25578150

[63] Aresta C Soranna D Giovanelli L Favero V Parazzoli C Gennari L . When to suspect hidden hypercortisolism in type 2 diabetes: A meta-analysis. Endocrine practice: Off J Am Coll Endocrinol Am Assoc Clin Endocrinologists (2021) 27(12):1216–24. doi: 10.1016/j.eprac.2021.07.014 34325041

[64] Tiryakioglu O Ugurlu S Yalin S Yirmibescik S Caglar E Yetkin DO . Screening for cushing’s syndrome in obese patients. Clinics (Sao Paulo Brazil) (2010) 65(1):9–13. doi: 10.1590/S1807-59322010000100003 20126340 PMC2815288

